# ‘Come together’—The Regulatory Interaction of Herpesviral Nuclear Egress Proteins Comprises Both Essential and Accessory Functions

**DOI:** 10.3390/cells11111837

**Published:** 2022-06-04

**Authors:** Sigrun Häge, Manfred Marschall

**Affiliations:** Institute for Clinical and Molecular Virology, Friedrich-Alexander-Universität, Erlangen-Nürnberg (FAU), 91054 Erlangen, Germany

**Keywords:** herpesviruses, human cytomegalovirus, regulation of viral replication, nuclear egress complex (NEC), NEC hook and groove proteins, functional properties, conditional expression, specialized functional aspects, efficiency of virus production, antiviral drug target

## Abstract

Herpesviral nuclear egress is a fine-tuned regulatory process that defines the nucleocytoplasmic release of viral capsids. Nuclear capsids are unable to traverse via nuclear pores due to the fact of their large size; therefore, herpesviruses evolved to develop a vesicular transport pathway mediating the transition across the two leaflets of the nuclear membrane. The entire process involves a number of regulatory proteins, which support the local distortion of the nuclear envelope. In the case of the prototype species of β-*Herpesvirinae*, the human cytomegalovirus (HCMV), the nuclear egress complex (NEC) is determined by the core proteins pUL50 and pUL53 that oligomerize, form capsid docking lattices and mediate multicomponent assembly with NEC-associated viral and cellular proteins. The NEC-binding principle is based on the hook-into-groove interaction through an N-terminal hook-like pUL53 protrusion that embraces an α-helical pUL50 binding groove. Thus far, the function and characteristics of herpesviral core NECs have been well studied and point to the groove proteins, such as pUL50, as the multi-interacting, major determinants of NEC formation and egress. This review provides closer insight into (i) sequence and structure conservation of herpesviral core NEC proteins, (ii) experimentation on cross-viral core NEC interactions, (iii) the essential functional roles of hook and groove proteins for viral replication, (iv) an establishment of assay systems for NEC-directed antiviral research and (v) the validation of NEC as putative antiviral drug targets. Finally, this article provides new insights into the conservation, function and antiviral targeting of herpesviral core NEC proteins and, into the complex regulatory role of hook and groove proteins during the assembly, egress and maturation of infectious virus.

## 1. Current Concept of Herpesviral Nuclear Egress Regulation

The virus family, *Herpesviridae*, includes a number of important human and animal pathogens causing a broad variety of symptoms of infection that include lethal courses, tumors and immunological disorders [1]. The type species of the three herpesviral subfamilies α-, β- and γ-*Herpesvirinae* are represented by herpes simplex virus 1 (HSV-1), human cytomegalovirus (HCMV) and Epstein–Barr virus (EBV), respectively. Among these, HCMV is a major human pathogen with worldwide distribution that is able to persist lifelong in its human host. While most of HCMV infections remain asymptomatic in the immunocompetent, severe or even life-threatening sequelae may occur in immunosuppressed individuals or immunonaive unborn and newborns. As a striking feature, HCMV-induced pathogenesis is widely determined by the magnitude of viral reproduction [2]. This means that specific cytomegaloviral pathogenic patterns in humans are mostly determined by active virus multiplication in the infected tissues and high viral load.

**Figure 1:**
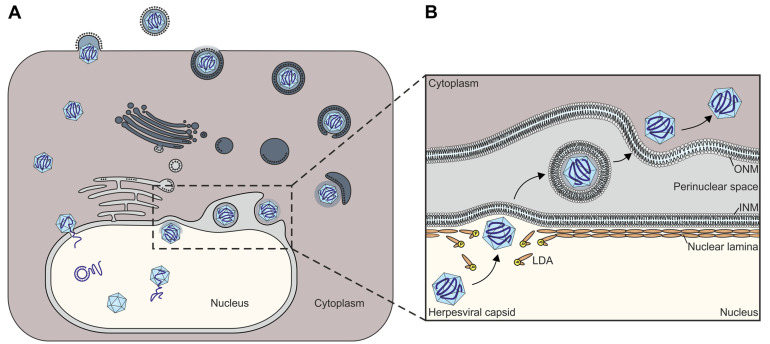
Prototypic replication and nuclear egress of herpesviruses. (**A**) During herpesviral replication capsids are transported to the inner nuclear membrane (INM), where the viral genome is released into the nucleus. In the nucleus, the viral gene replication and expression starts, and the newly synthesized genomes are packed into preformed capsids. The capsids are exported from the nucleus via the nuclear egress (**B**), and in the cytoplasm the virions finally mature and release the cell. (**B**) For the nucleocytoplasmic transport of the capsids, referred to as nuclear egress, the nuclear lamina has to be dissolved by phosphorylation (P) of lamins. At these lamina-depleted areas (LDAs), capsids bud at the INM into the perinuclear space. These primary enveloped virions then fuse with the outer nuclear membrane (ONM), releasing the capsid into the cytoplasm.

During lytic replication, the synthesis and encapsidation of the viral genomic DNA occurs in the nucleus, prior to the subsequent steps of maturation of infectious particles that proceed in the cytoplasm. Initially, during this sequence of events, the de novo synthesized viral genomes are packaged into preformed nuclear capsids, thus representing a central step in the herpesviral replication cycle. After this initial assembly step, the mature C capsids have to face a rate-limiting process, in that they need to leave the nucleus for further cytoplasmic maturation and the extracellular release of viral particles. However, since the nucleus is surrounded by the nuclear envelope (NE), composed of the inner and outer double membranes (INM and ONM), the nuclear pore complex (NPC) and the lamina network, viral capsid transition is limited. It is mostly due to the rigid proteinaceous network of the nuclear lamina that the NE serves as a highly selective barrier reserved for the transport of small-size or signal-labeled molecules [3]. Of note, particles larger than 40 nm are not able to be transported through NPCs [4]. Since the diameter of herpesviral capsids is approximately 130 nm [1], herpesviruses have emerged as a specific mechanism, the so-called regulated nuclear egress, to bypass this barrier in a finely coordinated manner (Figure 1). Thus, the nuclear egress is a crucial step during the late phase of replication and is conserved among α-, β- and γ-herpesviruses [5,6,7,8,9,10,11,12,13,14,15,16,17,18,19].

In our previous studies, we mainly focused on the HCMV-specific nuclear egress and the regulatory proteins involved. In the case of HCMV, nuclear egress is primarily regulated by the two heterodimerizing viral proteins, the transmembrane protein pUL50 and the nucleoplasmic pUL53, which colocalize at the NE, thereby defining the core nuclear egress complex (NEC). This core NEC recruits a number of viral and cellular NEC-associated factors, ultimately resulting in a multicomponent NEC, which then mediates the reorganization of the nuclear lamina and represents a docking platform for nuclear capsids. Thus, the regulated nuclear egress promotes the envelopment and transient de-envelopment of genome-packaged C capsids at the INM and ONM as a part of the multistep nucleocytoplasmic capsid transition. Interestingly, different to the understanding previously anticipated, the regulatory principle of herpesviral nuclear capsid egress is not unique to herpesviruses as illustrated by a number of molecular studies on cell biology. Obviously, this transport pathway also represents a specific mode of the cellular vesicle-mediated transport that is able to mediate the delivery of various types of cargo through the NE [8,13,20,21]. Thus, in many aspects, the herpesvirus-specific egress process is reminiscent of cellular vesicle-mediated transport [8]. This is particularly true for intracellular trafficking, during which the budding and scission of membrane vesicles is meditated by specialized proteins. Such proteins, for instance clathrin and functionally related factors, are able to induce cytosolic membrane curvature in order to extrude cytoplasmic vesicles. As another comparable feature, the cellular vesicle formation can be based on the invagination of the endosomal membrane as reported for the formation of multivesicular bodies of exosomes [22,23].

## 2. The Herpesviral Heterodimeric Core Nuclear Egress Complexes

### 2.1. Two Functionally Specialized Proteins Determine the Core of the Herpesviral NECs

**Figure 2:**
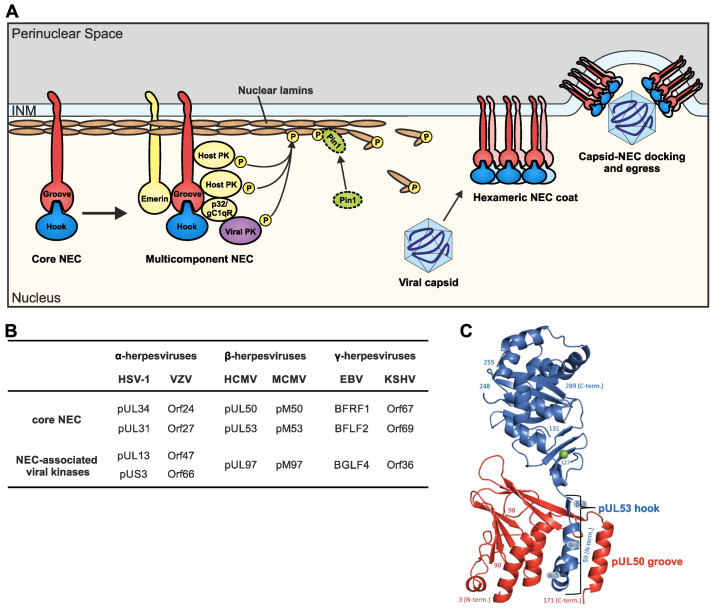
Schematic representation of the nuclear egress and the conserved herpesviral core NEC proteins. (**A**) The herpesviral core NEC is formed by two interacting viral proteins, the hook and the groove protein, which recruit several cellular and viral proteins, in particular protein kinases (PKs), resulting in the multicomponent NEC. At present, it is still unknown whether host PKs may replace the role of viral PKs (note that kinase-negative viral mutants are still moderately replication competent). The local disruption of the lamina network depends on site-specific phosphorylation (P) of nuclear lamins A/C and is mediated by PKs and the isomerase Pin1. The resulting lamina-depleted areas (LDAs) and the oligomerization of the core NEC heterodimers into hexameric rings facilitate the lamina transition and the budding of capsids through the inner nuclear membrane (INM; modified from [35]). (**B**) Conserved core NEC proteins and NEC-associated viral protein kinases of six herpesviruses (exemplarily, the NEC association was clearly validated for HCMV pUL97, and is likewise postulated for other herpesviral PKs): VZV, varicella zoster virus; KSHV, Kaposi’s sarcoma-associated herpesvirus. (**C**) Crystal structure of the HCMV-specific heterodimeric core NEC characterized by the hook structure of pUL53 (blue) binding into the groove structure of pUL50 (red); modified from [33].

One of the basic principles of cellular biology is given by the border line of the NE that represents a physical barrier separating the nucleus from the cytoplasm. As a characteristic feature of herpesviruses, the viral nuclear egress is a highly coordinated, multistep regulatory process. In this regard, the herpesviral NEC is a fascinating example of how a viral heterodimeric element (i.e., the core NEC) can provide a scaffold that hijacks host-specific functions including protein transport and intracellular trafficking [5,6,7,8,9,10,11,12,13,14,16]. Considering the specific case of HCMV, a central mechanistic determinant of nuclear egress is given by the multi-interacting pUL50, which is anchored to the nuclear membrane through its transmembrane domain (TMD). As a key point of functionality, pUL50, on the one hand, is able to recruit its nucleoplasmic binding partner, pUL53, to the nuclear rim by a hook-into-groove interaction (Figure 2A, left part). On the other hand, pUL50-recruited assemblies are also recruiting further NEC-associated proteins, in particular the viral kinase pUL97 [24] as well as cellular kinases like CDK1 [25], the prolyl cis/trans isomerase Pin1 [26] and a number of additional regulators [12,27]. Especially, these kinases play a crucial role during nuclear egress by the phosphorylation of the nuclear lamins. This site-specific lamin phosphorylation initiates a local reorganization of the nuclear lamina (lamina-depleted areas, LDAs), so that capsids ultimately attain access to the INM (Figure 2A, right part). Interestingly, it was also shown that pUL50 is phosphorylated by these NEC-associated protein kinases in a site-specific manner. However, our recent report illustrated that a mutagenesis-based suppression of these phosphorylation events did not impair viral replication [28]. As far as the conservation of NEC protein compositions are concerned, it should be emphasized that the heterodimeric core formed by two viral NEC proteins, as well as the NEC association of viral protein kinases, appear to be similar for all herpesviruses of the three subfamilies (Figure 2B). The composition of the entire multicomponent NEC, however, including further cellular proteins of the NE, additional host enzymes such as Pin1 or viral NEC-associated proteins, can profoundly differ among individual herpesviruses (reviewed in [12]; for murine cytomegalovirus (MCMV), see [29]; for HCMV, see [27]; for HSV-1, see [30]). 

### 2.2. The Hook-into-Groove Principle of Core NEC Heterodimerization

The specific mode for how the two herpesviral core NEC proteins interact with each other is defined by their unique structural features and has been designated as the hook-into-groove binding principle (Figure 2C). Both proteins are characterized by highly globular folds, comprising a combination of α and β secondary structural elements. The heterodimeric interaction is mediated by the hook structure of pUL53 (amino acids 59–87) binding into the groove structure (amino acids 1–209) of pUL50 (Figure 2C; [31,32,33]). The specific hook structure of pUL53 is characterized by two α-helices, also termed αN (amino acids 61–70) and αC (amino acids 72–81), followed by a β-sheet and the main globular domain. Of note, pUL53 contains a zinc-binding site, formed by three cysteine residues and a histidine (i.e., Cys106, Cys122, Cys125 and His 211), but the function of this classical zinc-binding site still remains elusive [31,33]. The groove structure of pUL50 is formed by four α-helices and an almost all-antiparallel β-sheet sandwich, embedding the hook structure of pUL53 [32,33]. This specific hook-into-groove interaction is based on mixed physicochemical properties. Besides the hook-into-groove interface, the complex comprises three additional major sites of interaction mediating the oligomerization of the pUL50-pUL53 heterodimers into hexameric rings. These three oligomer interfaces are important to connect (i) the two neighboring pUL53 proteins, (ii) the pUL50 of one dimer to pUL53 of a neighboring dimer and (iii) the two neighboring pUL50 proteins [34]. Although these higher NEC oligomer interfaces are conformational rather labile, the hexameric arrangements, associating to honeycomb-like lattices, provide an important basis for the formation of curved structures, required for the nuclear vesicle formation during the budding process [12,33,34].

## 3. Multiple Viral and Host Components Can Be Associated with Nuclear Egress Complexes

### 3.1. Herpesviral Multicomponent NECs Represent Assembly Platforms That Recruit a Variety of Nuclear Proteins with Regulatory Activities

The core NEC is associated with several viral and host components forming the multicomponent NEC. Thus far, the exact composition of this complex is poorly understood. However, in the case of HCMV, pUL50 seems to be the multi-interacting determinant mediating the formation of the multicomponent NEC, whereas the functional role of pUL53 still remains largely unresolved. However, there are experimental indications that pUL53 may be involved in the recruitment of the capsids to the NE [36], an initial finding that has to be further investigated in detail. Recently, a more specific analysis of pUL53 functional properties supported the point that pUL53 may be responsible for nuclear viral capsid–NEC recruitment. In addition, its binding to myosin Va was identified. Interestingly, further data led to the statement that pUL53 might not be important for capsid transition towards the nuclear periphery [37]. A very prominent host factor of the multicomponent NEC has been identified with the multi-ligand binding protein p32/gC1qR. First detected in association with the HCMV-specific kinase pUL97 [38], it became more and more clear that p32/gC1qR acts as a bridging factor that associates the regulatory important pUL97 activity to the NEC [27]. This NEC bridging function of p32/gC1qR, through direct contacts with pUL50 and pUL97, has been demonstrated by a number of different experimental approaches such as yeast two-hybrid screening, coimmunoprecipitation and in vitro assembly assays, pUL97 in vitro kinase assays, confocal imaging of the NEC nuclear rim, mass spectrometry-based proteomics and other methods [12,27,36,38,39,40,41]. In addition, the HCMV core NEC binding of p32/gC1qR has been assigned to the central globular domain, amino acids 100–358 of pUL50 [42]. Several further studies underlined the presence of p32/gC1qR not only in further β-herpesviral NECs [29] but also in α-herpesviral and γ-herpesviral NECs [30,43]. Additional host factors associated with herpesviral NECs include the following: emerin (a prominent integral NE component), protein kinase C alpha (PKCα), cyclin-dependent kinase 1 (CDK1), endophilin A2 (a regulator of membrane scission in clathrin-independent endocytosis), cis/trans isomerase Pin1 (a multifunctional phosphorylation-dependent prolyl isomerase), nuclear importins/NUPs (main transport factors of the NPC), torsin A (an ATPase of the ER and perinuclear space), RASCAL (a lamin B-associated regulatory factor), BiP (an ER-localized chaperone), lamins A/B/C (the main constituents of the nuclear lamina), lamin B receptor (LBR) and several other proteins (reviewed in [12]). 

### 3.2. Similarities and Differences between the Constituents of Herpesviral Multicomponent NECs

Comparing all herpesviral NECs analyzed so far, based on proteomics and functional analyses, the NEC-associated cellular proteins comprise the abovementioned typical lamina/NE proteins as well as various regulatory factors. Hereby, it is surprising that the differences in the overall composition of these multicomponent NECs appear to be quite pronounced, at least when comparing viruses derived from the different subfamilies of α-, β- and γ-herpesviruses, considering the fact that the basic features of NEC functionality and NE remodeling are very similar (Table 1). A plausible explanation for differences in the identified cellular NEC constituents is based on overlapping and possibly secondary functions exerted by nonidentical proteins in the different systems. Thus, it is highly suggestive that some NEC-associated factors, as particularly found for HCMV, may be substituted by different proteins fulfilling similar tasks in the NECs of other herpesviruses. Thus, an interesting feature of herpesviral NECs is that in terms of sequence, structural and functional properties, they likewise include both consistent as well as different properties. As seen from the aspect of sequence conservation, a gradual adaptation is seen for closely related herpesviruses compared to more distinctly related species belonging to different subfamilies (see Section 5.1, Ref. [35]). In the context of 3D structures, in particular concerning the hook-into-groove binding elements, very similar, if not identical, properties could be identified. However, the constituents contained within the multicomponent NECs may vary substantially among the individual herpesviruses, as discussed under Section 3.1. Finally, as a principle, the finely regulated overall functionality of viral nuclear egress is almost identical among all herpesviruses (Table 1). In essence, it is most plausible to consider that those features that make the differences among herpesviruses are widely balanced and compensated by alternative factors and regulatory steps in the nuclear egress process.

## 4. Main Functionality and Regulatory Roles Shared by Herpesviral NECs

### 4.1. The Key Role Played by Herpesviral NECs during the Viral Lytic Replication Cycle

The process of herpesviral nuclear egress is embedded into a long sequence of regulatory events within the nuclear phase of the viral lytic replication cycle (Figure 1). After the nuclear import of viral genomes, genomic replication, protein synthesis and capsid assembly, the subsequent processes of genome encapsidation, ongoing capsid nuclear egress and primary envelopment directly proceed. Interestingly, as specifically addressed for the regulatory procedures of HCMV replication, a number of viral regulators involved in one of these stages may also exert further supportive activity in other stages, as many of these are multifunctional [44]. Thus, it is not surprising that the sensitive proteomics studies demonstrated a detectability of viral regulatory proteins in not only one distinct multiprotein complex but in other complexes of these interlinked replicative stages as well [45,46,47,48]. Against this background of knowledge, it is not surprising that the herpesviral core NEC proteins also have secondary activities (e.g., HSV-1 pUL34 [49]; HCMV pUL50 [50,51]; EBV BFRF1 [52,53,54]), and the multicomponent NEC may be figuratively seen as a Swiss army knife, having incorporated a number of NEC-specific regulatory activities [12,13,55,56,57]. Considering the numerous studies on NEC functionality performed so far, four main steps in the regulation of herpesviral nuclear egress can be defined as follows: (i) the coordinated core NEC heterodimerization and oligomerization to provide a basic scaffold, (ii) the formation of a multicomponent NEC that carries multifold activities, (iii) the phosphorylation-induced reorganization of the nuclear lamina and (iv) the NEC docking and nuclear membrane budding of viral capsids. In particular, the involvement of protein kinases in these events appears important for regulation. Not only has the NEC association of herpesviral protein kinases been demonstrated, but also the direct or indirect involvement of host kinases has been strongly suggested, such as CDK1, additional CDKs, PKCα, PKCδ, ERK1/ERK2, and probably even more [15,16,58,59,60,61,62]. Site-specific phosphorylation of the nuclear lamins by NEC-associated kinases has been identified as the main trigger of massive rearrangements of the NE, consequently leading to the formation of lamina-depleted areas (LDAs), the sites where viral nuclear capsids gain access to the INM [63,64,65]. Additional events of NE reorganization, specifically the formation of a hexameric NEC coat and patch-like lattice that appears to serve as a platform for capsid docking, allow for the budding of intranuclear capsids into the perinuclear space [12]. Hitherto, mainly, the regulation of the nuclear egress of some exemplary herpesviruses has been mechanistically elucidated including the identification of NEC-associated proteins [12,27]. Additionally, structural investigations revealed wide-ranging similarities between α-, β- and γ-herpesviral NECs [11,31,32,66,67,68,69,70,71]. So far, the 3D structures of five different herpesviral core NECs have been crystallographically determined by independent groups [22,31,32,33,70,72]. The multiple methods of interaction between viral core NEC proteins and host cell factors, including their functional impact, is a key issue in understanding this essential step in the herpesviral replication cycle and in virus-induced pathogenesis. 

### 4.2. Comparative Aspects of Herpesviral Nuclear Egress and Cellular NE-Specific Processes

An important step during the nuclear egress is the partial disruption of the nuclear lamina and the reorganization of the NE. Through the activity of pUL97 in the case of HCMV infection, the site-specific phosphorylation of lamin A/C at Ser22 generates a binding motif for Pin1 [26]. Lamin-associated Pin1 obviously isomerizes peptidyl-prolyl peptide bonds [73,74], resulting in conformational changes of the lamins and, thus, in the locally distinct disassembly of the nuclear lamina (LDAs). Such disassembly events are not only observed in herpesvirus-infected cells but are also required in cellular processes such as mitosis, in the latter case referred to as nuclear envelope breakdown (NEBD) [75,76]. While the NEBD during mitosis is initiated by site-specific phosphorylation of lamins mediated by cellular kinases, namely, the cyclin-dependent kinase 1 (CDK1) and the protein kinase C (PKC) [77,78,79], herpesviruses probably hijacked this cellular mechanism using the cellular but also their virus-encoded protein kinases to induce lamina depleted areas (LDAs; [24,26,38,41,80,81,82,83]. The conserved herpesviral serine/threonine kinases (Figure 2B) are designated as CDK orthologs, sharing similar functions among themselves and among CDKs such as nuclear lamin phosphorylations [55,59,84,85,86,87,88]. Functional replacement between herpesviral kinases has been demonstrated with specific examples [89,90,91,92]. Consequently, site-specific Ser22-phosphorylated lamins are recognized by the cellular peptidyl-prolyl cis/trans isomerase Pin1, which isomerizes the lamins towards conformational reorganization and, thus, disassembles the lamina network to provide the capsids access to the INM [26,41,93]. Concerning the membrane-specific effects of the nuclear egress pathway, several highly valuable model systems have been established, in particular representing α-herpesvirus infections [6,7,94,95,96]. While the step toward the distinct recruitment of viral capsids to the NE is still insufficiently understood, several studies investigated the budding process in which core NEC proteins are involved by inducing membrane fission. As based on the available 3D crystal structures of five herpesviral core NEC pairs, spanning the three subfamilies [22,31,32,33,70,72], the concepts that are basic for understanding herpesviral primary envelopment have been further developed. For HSV-1 and HCMV, it has been demonstrated that their heterodimeric core NECs can oligomerize to form hexameric rings, which may associate to honeycomb-like lattices and, thus, enable a membrane curvature and, subsequently, the budding of capsids [33,72]. In addition, specific in vitro experiments demonstrated that HSV-1 core NEC proteins are sufficient to induce a negative membrane curvature, which is an essential prerequisite for viral scission and budding [64,96].

## 5. The Correlation between Herpesviral Core NEC Primary Sequences, Structural Conservation and Binding Properties

### 5.1. What Is the Degree of Conservation between the Primary NEC Amino Acid Sequences?

The conservation of primary sequences has to be considered in the context of structural and functional properties. To date, the crystal structures of five core NECs belonging to all three herpesviral subfamilies, α, β and γ, have been resolved. Aa a main finding, it turned out that despite low amino acid sequence identities between the NECs of HSV-1, pseudorabies virus (PrV), VZV, HCMV and EBV, the structural features and the principle of hook-into-groove interaction are highly conserved [11]. Moreover, the main functionality of core NECs is also specifically conserved among herpesviruses and, thus, the multi-ligand-binding core NEC proteins might serve as a particularly suitable target for antiherpesviral drugs, either in a selective or a broad-spectrum inhibitory manner. 

**Figure 3:**
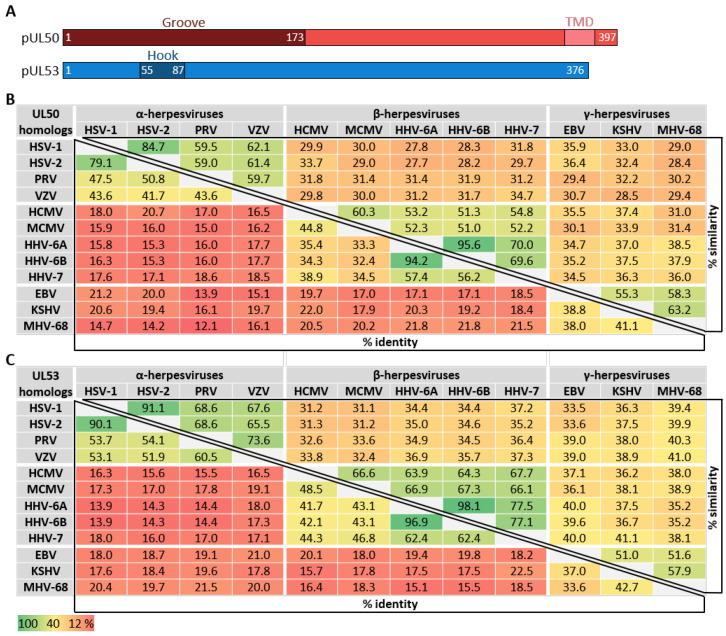
Amino acid sequence identities and similarities in % of pUL50 and pUL53 homologs of nine human and three animal herpesviruses. (**A**) Schematic depiction of pUL50 and pUL53. Amino acid positions are represented by numbers; TMD, transmembrane domain. (**B**) Homologs of HCMV pUL50 (groove proteins). (**C**) Homologs of HCMV pUL53 (hook proteins). UniProt accession numbers: HSV-1, P10218 and P10215; HSV-2, P89457 and P89454; VZV, P09280 and P09283; PRV (SuHV-1), T2FKZ7 and G3G955; HCMV, P16791 and P16794; MCMV (MuHV-1), D3XDN8 and D3XDP1; human herpesvirus 6A (HHV-6A), P52465 and P28865; HHV-6B, Q9QJ35 and Q9WT27; HHV-7, P52466 and P52361; EBV, P03185 and P0CK47; KSHV (HHV-8), F5HA27 and F5H982; murine gammaherpesvirus 68 (MHV-68/ MuHV-4), O41968 and O41970.

**Figure 4:**
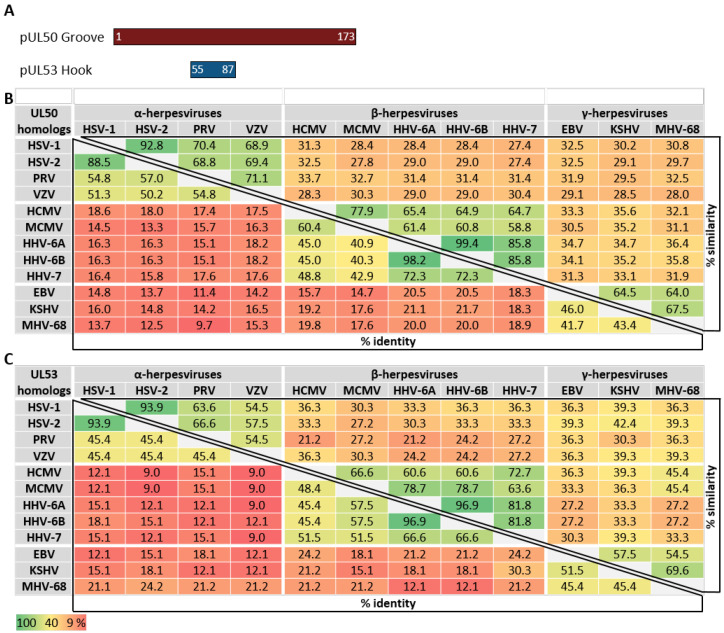
Amino acid sequence identities and similarities in % of the pUL50 groove- and pUL53 hook-specific segments of nine human and three animal herpesviruses. (**A**) Schematic depiction of the pUL50 groove and pUL53 hook. Amino acid positions are represented by numbers. (**B**) Homologs of HCMV pUL50 (groove proteins). (**C**) Homologs of HCMV pUL53 (hook proteins).

As far as the conservation of primary sequences of the core NEC proteins is concerned, a stepwise graduation of levels of conservation was recently reported [11,32,35]. Members of the subfamilies α-, β- and γ-*Herpesvirinae* showed marked differences in sequence characteristics. When comparing the entire amino acid sequences of core NEC proteins regarding the levels of identity and similarity, a very poor conservation can be recognized (Figure 3). In general, within individual herpesviral subfamilies, the level of conservation was found to be higher than between different subfamilies. With very few exceptions, the degree of conservation, in both the percentage of identity and percentage of similarity, is gradually decreasing along the increasing distance of virus species relationship. Moreover, it was considered as a striking feature that even the comparison between the sequences determining the structures involved in the interaction, the hook and groove segments (Figure 4), did not show a substantially higher degree of conservation compared to the entire proteins. In both cases of comparison, either between entire sequences or the specific hook and groove segments, the analyzed amino acid identities within α-herpesviruses ranged between 41.7–97.1% (entire UL50) or 50.5–88.5% (UL50 groove) and 51.9–90.1% (entire UL53) or 45.4–93.9% (UL53 hook). Within β-herpesviruses, they ranged between 32.4–94.2% (entire UL50) or 40.3–98.2% (UL50 groove) and 41.7–96.9% (entire UL53) or 45.4–96.9% (UL53 hook); within γ-herpesviruses, they ranged between 38.0–41.1% (entire UL50) or 41.7–46.0% (UL50 groove) and 33.6–42.7% (entire UL53) or 45.4–51.5% (UL53 hook) (Figure 3 and Figure 4). This may indicate that the sequence conservation is not specifically restricted to the NEC hook-into-groove interaction segments, but it is also seen for the entire sequences of the globular domains and even additional functionally important regions such as TMDs, nuclear localization signals (NLS), and binding interfaces of NEC-associated proteins. 

### 5.2. How Is the Correlation between NEC Sequences, Structures and Binding Properties?

**Figure 5:**
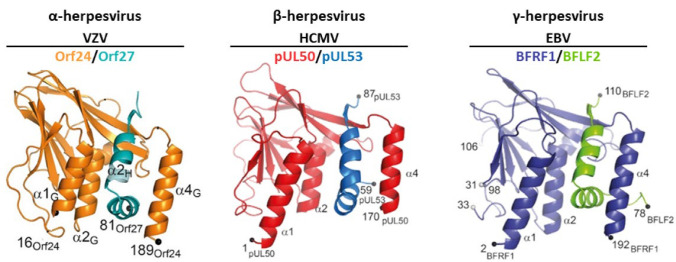
Structural conservation of the VZV, HCMV and EBV core NECs. Comparison of currently available 3D core NEC crystal structures of VZV, HCMV and EBV showing the hook-into-groove binding interface; modified from [32,70].

Interestingly, the finding that the structural domains of core NEC proteins are highly conserved stands in contrast to the very limited sequence conservation, and even the key amino acids within NEC-binding interfaces are not strictly conserved. Moreover, although a high functional consistency was found when regarding the basic role of NECs in nuclear egress, a marked specification was identified in regard of the limited cross-viral binding properties of core NEC pairs. The available structures of five herpesviral core NEC pairs [22,31,32,33,70,72] show almost full conservation and basically only vary in the lengths of the structural elements (Figure 5). In addition, the available published crystal structures and reports on several other NEC properties [11,12] showed that the principle of the hook-into-groove interaction is identical in the so far investigated herpesviruses. Regarding the more functional aspects of the core NEC, which will need further research for a comprehensive understanding, there is no doubt regarding the fact that the final outcome of the egress mechanism is conserved [5,6,7,8,9,10,11,12,13,14,15,16,17,18]. 

**Figure 6:**
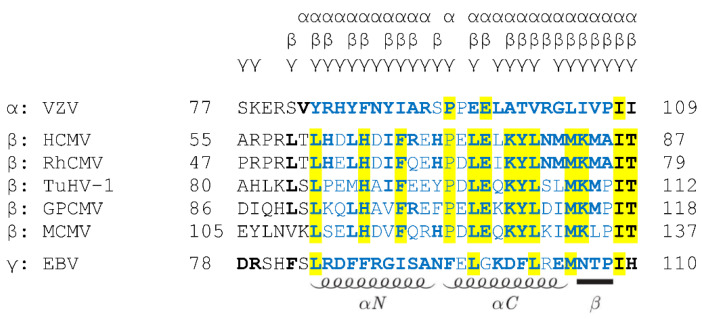
Lack of a simple correlation between the contact amino acids of hook-into-groove interfaces (marked by letters on top) and the amino acids strictly conserved across α-, β- and γ-herpesviral nuclear egress proteins (marked by yellow shading). Amino acid sequence alignment of the hook sequences of several human and animal herpesviral pUL53 homologs. Conserved amino acids are shaded in yellow (i.e., fully conserved between the analyzed β-herpesviruses), the hook-determining amino acids are in blue, and the elements of secondary structure are depicted schematically below the alignment. The hook-into-groove contact residues of VZV, HCMV and EBV are in bold and additionally labeled by the letters α, β and γ on top of the alignment. Amino acids of the animal β-herpesviruses identical to the contact residues of HCMV are also in bold. The numbers represent amino acid positions. UniProt accession numbers: VZV, P09283; HCMV, P16794; rhesus cytomegalovirus (RhCMV), O71122; herpesvirus tupaia (TuHV-1), Q91TN5; guinea pig cytomegalovirus (GPCMV), U6H9V2; MCMV, D3XDP1; EBV, P0CK47.

Another striking aspect of NEC characteristics is the lack of a simple correlation between the contact amino acids of hook-into-groove interfaces (Figure 6, marked by letters on top [32,70]) and the amino acids strictly conserved across α-, β- and γ-herpesviral nuclear egress proteins (Figure 6, marked by yellow shading). While most of the contact amino acids of hook elements (bold) (i.e., responsible for the NEC hook-into-groove binding) were found to be identical between the β-herpesviral sequences (yellow), only very few of these were seen in an overall conservation across α-, β- and γ-herpesviral sequences (Figure 6, compare bold with yellow). This may indicate that the mechanism of an evolutionary conservation of structural properties mostly did not include a conservation of primary sequences [11]. Nevertheless, the high structural identity and the conserved mechanism of nuclear egress led to the assumption that cross-viral interactions between the core NEC proteins of different herpesviruses should be possible. However, the analysis of such cross-viral nonautologous core NEC interactions demonstrated that cross-viral interactions and nuclear rim colocalizations were only possible for core NEC combinations within individual subfamilies, indicating that the structural similarity is not the only decisive factor [35]. Together with the previous data indicating that the sequences are stronger conserved within the same subfamily than between different subfamilies, the subfamily-spanning interaction patterns hinted to the possibility that the amino acid sequences may define virus-specific binding properties of NEC proteins. Moreover, these interaction analyses also confirmed findings of previous cross-complementation studies. Two earlier studies have shown that the core NEC proteins of EBV and KSHV can cross-interact and remodel the nuclear membrane indicating that a cross-complementation between γ-herpesviruses is possible [97,98]. A very similar finding was also obtained for the β-herpesviruses, whereby recombinant MCMVs, in which the ORF M50 or M53 was either replaced by the HCMV ORF UL50 or UL53 gene homolog, respectively, were investigated. Both virus variants were able to replicate and did not significantly differ from the wild-type (WT) [99]. This functional replacement of M50 in MCMV by UL50 was also confirmed by our group using an in vivo mouse model [35]. A comprehensive study by Schnee et al. [99] not only investigated the cross-complementation properties within individual herpesviral subfamilies but also between different subfamilies. The authors replaced the M50 or M53 of MCMV by the respective homologs of HSV-1, PrV, MHV-68 and EBV, resulting in subfamily-spanning recombinant viruses. However, none of these replacements were functional in the viral background. A very recent study of the α-herpesviruses HSV-1 and VZV showed that the replacement of UL34 by Orf24 in HSV-1 did not lead to a replication-competent virus recombinant, although on the protein levels, the pUL31 and Orf24 showed positive interaction [19]. However, a minimal selection for viral replication in cell culture revealed some functional variants which acquired additional mutations in Orf24, ICP22, ICP4 and US3, in order to replicate in an almost WT-like manner. The authors of this study provided two possible explanations for why this cross-viral core NEC was not functional. On the one hand, they discussed that Orf24 was not able to recruit other viral proteins of the multicomponent NEC, and on the other hand, it was considered possible that the hexameric ring formation was not provided due to the sequence differences of the two herpesviral core NEC proteins. Additionally, the aspect of core NEC functionality was further investigated using the ΔUL50 virus and Orf24-, pM50- and BFRF1-complementing cells (M.M., S.H. et al., unpublished data). However, none of the complementing cell lines were able to completely rescue the ΔUL50 phenotype, suggesting that there is no perfect cross-viral complementation achievable even within NEC proteins of identical subfamilies. Interestingly, it is becoming increasingly apparent that either the minor differences within the lengths of the α-helices, β-strands and the loop segments of the hook and groove structures may represent factors defining the binding specificity of each core NEC, or distinct positions of the primary amino acid sequences may play a crucial role in binding specificity.

## 6. Mutational Analysis Focusing on Specific Herpesviral NEC Functions

### 6.1. Phenotypical Features of Herpesviral Core NEC Proteins Determined by Viral Deletion Mutants

**Figure 7:**
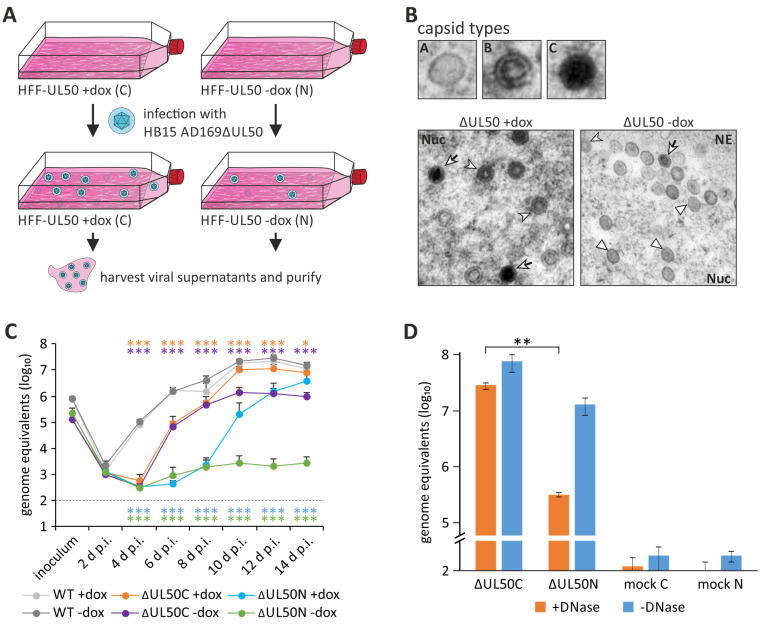
Generation and the phenotypical features of the ∆UL50 virus. (**A**) Schematic depiction of the purification of ∆UL50 particles. HFF-UL50 cells, either dox-induced (+dox) or uninduced (−dox) were infected with HCMV ∆UL50. Viral supernatants were harvested and purified, ∆UL50 particles derived from pUL50-complementing conditions were named ∆UL50C and from non-complementing conditions ∆UL50N. (**B**) Transmission electron microscopy (TEM) ∆UL50C-infected HFF-UL50 cells, either induced or uninduced, reveals a strong accumulation of immature A capsids (white triangle) and reduction of mature C capsids (white arrowhead with black tail) under the non-complementing condition; white arrowhead, B capsids. (**C**) Replication kinetics of the ∆UL50C and ∆UL50N particles on complementing or non-complementing cells showing a strong impairment of replication efficiency in the absence of co-expressed pUL50. Statistical significance of the values of genome equivalents was calculated by ANOVA (*, *p* ≤ 0.05; **, *p* ≤ 0.01; ***, *p* ≤ 0.001) for 2–19 d p.i. in relation to WT (mean value of WT +dox and WT −dox). (**D**) Comparison of viral genome levels in ∆UL50C and ∆UL50N particles by DNase treatment revealing strongly reduced levels of packaged viral genomes in the ∆UL50N particles. Statistical significance was calculated by the Student’s *t*-test (**, *p* ≤ 0.01) (modified from [28,103]).

There have been controversial discussions on whether herpesviral NEC proteins may generally be categorized into proteins having an essential role during viral replication or whether they can be dispensable under specific conditions. In any case, the absence of one of the two core NEC proteins, which are definitely important based on their conservation in functional and structural aspects (Figure 1B; [22,31,32,33,70,72]), leads to a reduction in the viral reproduction efficiency and progeny titers in a drastic way [66,67,68,69,71,100,101,102]. While there are several studies regarding the essentiality of the two core NEC proteins of HSV-1, PrV, MCMV and EBV, this aspect has only rarely been examined for HCMV. Many studies investigated the functionality of the HCMV core NEC, particularly with regard to the interactions forming the multicomponent NEC and the regulation of the nuclear egress. However, the question whether the two core NEC proteins are absolutely essential for viral replication has not been answered yet. Hence, our recent study focused on the characterization of the ΔUL50 virus, a recombinant HCMV lacking the ORF UL50 [28,103]. Surprisingly, this virus, as reconstituted on pUL50-complementing cells (ΔUL50C) and used as a stock virus for further infection experiments, was found impaired but not completely blocked in replication as seen with non-complementing cells. Interestingly, we detected a strong decrease in the production of mature C-type capsids in the absence of pUL50 expression, with a nuclear accumulation of immature A-type capsids (Figure 7). In our earlier reports, the ΔUL50 particles had commonly been produced in pUL50-complementing cells. However, on the basis of current knowledge that pUL50 is also packaged into virions, at least at very low amounts [104], the previously used ΔUL50 particles may not have completely missed pUL50. For this reason, in continuation of the studies, we produced two different versions of ΔUL50 particles: those either generated in pUL50-complementing cells (ΔUL50C) or in non-complementing cells (ΔUL50N; Figure 7A) [103], the latter, of course, obtained in much lower quantities. This comparison was made to gain deeper insight into functional details of pUL50 and the range of its regulatory roles exerted during the entire HCMV replication cycle. An important and basic finding was that the ΔUL50N particles, derived from non-complementing cells, exhibited a substantially stronger replicative defect than ΔUL50C particles (Figure 7B). This defect became even more apparent by performing a gradient purification in which no virion fraction was detectable in the case of the ΔUL50N virus. Remarkably, the proteomic composition of the noninfectious ΔUL50 particles (NIEPs of ΔUL50C and ΔUL50N) did not differ to a relevant extent. However, several experiments confirmed that the packaging of viral genomes into the capsids was massively disturbed in the absence of pUL50 (Figure 7D). This also explained the lack of an infectious virion fraction upon ΔUL50N gradient purification. Consequently, while the ΔUL50C particles were still able to infect non-complementing cells, albeit to a lesser extent, infection with ΔUL50N particles was almost completely negative under these conditions. Thus, as a central finding, the ΔUL50N particles lacked the normal portion of C-type capsids and encapsidated, infectious virions (Figure 7B). In conclusion, the experiments demonstrate that pUL50 is not completely essential for nuclear egress, per se, but obviously has an important regulatory role in viral maturation, especially in the packaging of viral genomes. 

### 6.2. Regulated Viral Nuclear Egress Compared to Nuclear Envelope Breakdown (NEBD)

The herpesviral egress is required to overcome the physico-chemical barrier of the NE (represented by nuclear lamina, INM, ONM and NPCs). This nucleocytoplasmic capsid transport is a specifically regulated process as described in chapter 1. In comparison to this, the cellular nuclear envelope breakdown (NEBD) is a well-studied process that regulates the disassembly of the nucleus during eukaryotic mitosis [75,76]. A partial NEBD, however, as occurring in the process of mitosis, is characterized by large gaps in the NE due to a phosphorylation-dependent disintegration of NPCs and lamins. These phosphorylation events are mediated by mitotic kinases, mainly CDK1 and PKC. Of note, it has been shown that both types of kinases and, in addition, also other cellular as well as virus-encoded protein kinases are involved in the nuclear egress of herpesviruses [25,81,105,106,107]. These findings indicate that herpesviruses may have captured this cellular mechanism. Another hint is the ESCRT apparatus, which repairs and remodels the NE after mitosis and also seems to be involved in herpesviral replication [9,75,76,108,109,110]. Thus, the core NEC proteins may activate and optimize the mitotic NEBD to use this mechanism in a more specific and efficient way for herpesviral replication and hence, can rely on this less efficient mitotic NEBD when the functionality of the core NEC proteins is disturbed. Moreover, it is noteworthy to mention that in addition to herpesviruses, several other viruses have to overcome this physico-chemical barrier of NE to enter or exit the nucleus and, hence, remodel the NE, albeit in various ways [111,112,113]. For example, the simian virus 40 (SV40) restructures the NE by the activation of specific caspases resulting in a direct cleavage of lamins and lamin-associated proteins, consequently hijacking the apoptosis-specific mechanism to mediate NEBD, but without inducing the complete pathway of programmed cell death [113]. 

However, the results obtained so far do not provide an insight into the exact mechanism in which way pUL50 contributes to the maturation of infectious virions. The statement which can be made by now is that, in the absence of pUL50, the level of encapsidated viral genomes is lower, suggesting that the packaging of genomes into preformed B capsids is impaired. In general, after capsid assembly, the portal complex interacts with the terminase complex, composed of several subunits [14,114,115,116,117]. This complex recognizes specific Pac sequences of the viral genomes and precisely cleaves the concatemers, leading to the packaging of one complete genome per capsid through the portal channel. During this packaging process, the scaffold, which is incorporated and characterizes B capsids, is disassembled by the protease, resulting in mature C capsids [94]. Until now, it is not known exactly where this packaging occurs in the nucleus, but most probably in direct or close proximity to the replication compartments as all the proteins involved in this process accumulate there. Since pUL50 is anchored in the NE by its TMD and hence, physically separated from replication compartments, this raises questions regarding a function during the genome packaging. A plausible explanation relies on the concept that the herpesviral NEC appears to act as a quality control of nuclear egress, selectively transferring mature C capsids through lamina-depleted areas of the NE [7,118,119]. Thus, a disturbance of this function would either lead to a release of immature A- and B-type capsids into the cytoplasm, or to a retention of mature C capsids in the nucleus. This mirrors the result of the mass spectrometry analysis of the study, in which a large number of NIEPs was received, representing enveloped B capsids. Moreover, this would also explain why herpesviruses do not simply use the cellular NEBD, releasing any nuclear content, but have developed a specific egress mechanism to achieve a very selective and efficient transport.

### 6.3. The Essential and Non-Essential Functional Properties of Herpesviral Core NECs

As far as the essential role of the HCMV core NEC proteins for viral replication is concerned, this has not really been evaluated in greater detail so far. For the two α-herpesviruses HSV-1 and PrV, the issue has been specifically addressed. Data strongly suggested that pUL31 of HSV-1 and pUL31 of PrV (the homologs of HCMV pUL53) are not absolutely essential for nuclear egress [66,67,68]. Indeed, the deletion of ORF-UL31 from the HSV-1 or PrV genomes did not lead to a complete block of viral replication, but instead to some relative decrease in viral loads and a partial deficiency in the production of enveloped particles. This phenotype could be restored by the use of pUL31-complementing cells, and a very similar finding was obtained for the deletion of ORF-UL34 (the homolog of HCMV ORF-UL50) [69,71,102]. However, similar to the present HCMV study, there was not a complete block of HSV-1 and PrV replication. A more surprising result was obtained by an extensive passaging of the PrV ORF UL31 or UL34 deletion mutants, which led to functional variants, termed pass mutants, almost producing WT titers [118,120]. In both cases, similar mutations in various viral proteins emerged and a virus-induced disintegration of the NE, a NEBD, was observed. Furthermore, it was also shown that the NEBD releases immature and mature capsids into the cytoplasm, suggesting that the core NEC also functions as a quality control checkpoint [118]. This aspect is highly consistent with the findings on HCMV ΔUL50 in the present study. Moreover, further experimental indications pointed to mitosis-related processes possibly involved in this herpesvirus-induced NEBD. In the case of EBV, deletion mutants in the coding sequences for nuclear egress proteins BFRF1 and BFLF2 were also described [100,101]. In both cases of deletion, a low-level replication of the recombinant viruses was demonstrated, specifically showing strong defects in the efficiency of nuclear egress and primary envelopment. Specifically, for the BFRF1-KO virus, the reduction in viral titers was due to the sequestration of EBV nucleocapsids in the nuclei of virus-producing cells [100]. For the ΔBFLF2 virus, in comparison, not only a defect in the nucleocytoplasmic egress was observed but also a defect in genome encapsidation [101]. As a more specific approach to gain insight into cytomegaloviral NEC proteins, two studies investigated the deletion of either ORF-M50 or ORF-M53, the core homologs of MCMV [121,122]. Both mutants of MCMV, either carrying a deletion of ORF M50 or M53, were not able to be reconstituted, but could be rescued by co-expressed pM50 or pM53, thus indicating that both core NEC proteins are actually essential for MCMV replication, at least under these experimental conditions. In summary, the studies do not provide a fully consistent picture about the essentiality of herpesviral core NEC proteins, but all of them demonstrate that viral replication is massively impaired in the absence of one of the proteins. 

Despite the intense research on NEC-specific topics during the last decades, there are still gaps in the current knowledge. These are mainly based on the fact, that the individual core NEC proteins have been characterized not only as structural NEC components, but rather as multifunctional viral regulators. However, the individual functional characteristics have not been deciphered yet. Thus, it will be pivotal for an overall understanding of herpesviral nuclear egress to further study the underlying functional details. These may include the question of nuclear core NEC–capsid interaction, the role in genome encapsidation, specific activities in nuclear trafficking, the triggers of host factor recruitment and additional aspects. In particular, the recently recognized suitability of the NEC as an antiviral target should be moved even stronger into the center of investigations. 

## 7. Validation of the NEC as a Unique Target of Anti-Herpesviral Therapy

### 7.1. Current Approaches and Proof-of-Concept That Support the Exploitation of the Herpesviral NEC as an Efficient Drug Target

**Figure 8:**
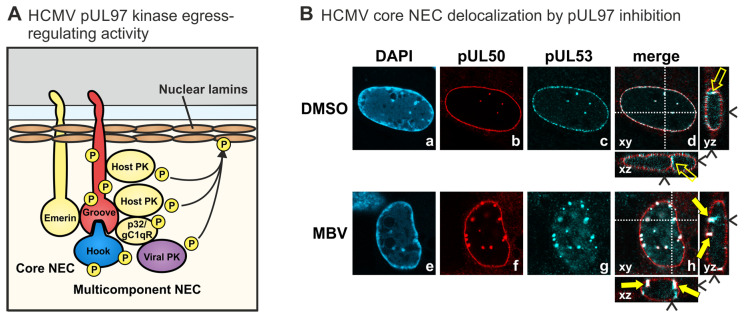
(**A**) The HCMV-encoded protein kinase pUL97 exerts a nuclear egress-regulating activity through the phosphorylation of nuclear lamins and additional proteins of the core and multicomponent NEC, including the hook and groove proteins themselves. (**B**) The HCMV core NEC undergoes a microscopically visible delocalization upon treatment with pUL97 inhibitors such as MBV or others. Specifically, treatment of HCMV-infected cells with the pUL97 inhibitor MBV results in intranuclear aggregates of pUL50 and pUL53. HFFs infected with recombinant HCMV AD169-GFP UL50-HA and treated with MBV (5 µM) or DMSO at 74 and 94 h post-infection (h p.i.). Cells were fixed at 96 h p.i. and subjected to indirect immunofluorescence analysis, and the subcellular localization of viral pUL50 and pUL53 was detected by recording confocal z-series. Representative images of a single focal plane (xy) were depicted for individual stainings, and xz and yz axes were depicted for merged images. White dotted lines and black arrowheads, optical section through the z stack (xy) or the focal plane (xz and yz); yellow-filled arrows, intranuclear aggregates of pUL50/pUL53 colocalization; yellow open arrows, transnuclear transport channels common in human fibroblasts (modified from [42]).

The herpesviral NEC has attracted the deep interest of researchers, since it represents a regulatory key position of viral replication and a putative target for novel antiviral strategies. Specifically on the basis of the data defining 3D structures according to NEC protein crystallization, the identification and development of a novel type of NEC-targeted drugs may now be realized. The validation of the NEC as an efficient target for small molecules exerting antiviral activity, has been achieved by several ways. These experiments included deletion and replacement mutants in NEC binding studies, recombinant viruses, experimental knock-down models, inhibitory peptides, NEC-directed small molecules, and some more [42,103,123,124,125]. A key point in this direction may have been the screening analysis using the Prestwick Chemical Library^®^ that contains a selection of pharmacologically valuable compounds and approved drugs [123,124]. The results of these studies provided a proof-of-concept that an inhibitory compound such as merbromin (MBM) that prevents the formation of the HCMV core NEC has the potential to block further processing of nuclear egress, eventually resulting in an intranuclear trapping of viral capsids. This concept of a nuclear entrapment of viral capsids has also been substantially supported by the studies using viral mutants carrying defects in the core NEC proteins, such as HCMV ∆UL50 (see Section 7.1). It appears highly promising that direct-acting antivirals (DAAs) that interfere with this viral replicative step of central importance may exert high efficacy and antiviral potency. The mechanism of NEC-directed antiviral activities has been investigated in our earlier studies including confocal imaging [25,28,42,124]. In these analyses, the drug-mediated effect onto the HCMV core NEC could be assessed in the form of a disturbed nuclear rim localization of viral pUL53 (Figure 8). Under the treatment with an NEC-active compound, the typical nuclear rim localization of egress protein pUL53 shows speckled patterns of delocalization in the form of intranuclear pUL53 aggregates (Figure 8A,B). Interestingly, this phenomenon of nuclear rim delocalization is not only found with core NEC-blocking small molecules such as MBM, but also with inhibitors of NEC-associated protein kinases such as maribavir (MBV; Figure 8B). MBV/Livtencity^TM^ is a selective inhibitor of the viral kinase pUL97, which exerts an important egress-regulating function in that it phosphorylates nuclear lamins as well as the core NEC proteins themselves and other NEC-associated factors (Figure 8A) [126]. This finding indicates that a block of viral NEC functionality may be pharmacologically achieved through several different mechanistic modes of NEC inhibition. Moreover, the NEC-directed antiviral activity might principally be realized in both ways, either in a virus-specific or in a broad, pan-herpesviral manner. This aspect can be considered from two different points of view. As far as the structural conformity of the core NEC is concerned, one might see a chance to generate a broadly active NEC-directed inhibitor. On the other hand, the highly specific requirements towards a sterical hindrance of such small molecules have to be taken into closer account. Drug-target docking may be based on very specific interaction of the drug with individual amino acids, forming a binding pocket or a finely structured microcanyon, which may argue against a broadness of inhibitory activity. This aspect favors the idea that a virus-specific mode of NEC inhibition appears more probable to be achieved. Thus, further proceedings in NEC-directed drug research may answer these questions. 

### 7.2. The Mode of NEC-Directed Antiviral Drug Activity Achieved through Several Defined Mechanistic Principles

**Figure 9:**
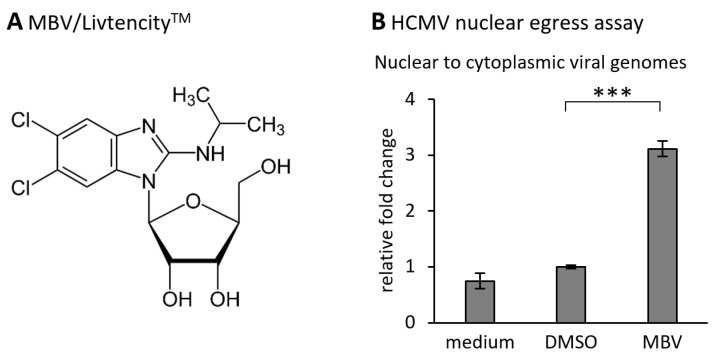
Maribavir (MBV) represents a newly approved anti-HCMV drug with nuclear egress-blocking activity. (**A**) Chemical structure of the benzimidazole riboside MBV. (**B**) The mode of antiviral activity of MBV was determined by a quantitation of viral encapsidated genomes [128]. The virus-specific qPCR-based measurements were performed after cellular fragmentation, i.e., using nuclear versus cytoplasmic fractions, using the recently established nuclear egress assay [125]. The significance between DMSO and MBV was calculated by Student’s *t*-test (*** *p* < 0.001).

Seen from the concept of a novel type of NEC-directed drugs, such inhibitory activity may be realized through a variety of different fine-mechanistic ways. An NEC-directed drug may be characterized by (i) the blocking of core NEC formation, i.e., through sterical hindrance of NEC protein interaction, (ii) the drug-mediated interference with activities of the multicomponent NECs such as site-specific phosphorylation, i.e., through inhibitors of NEC-associated herpesviral or cellular kinases, (iii) the inhibition of additional NEC-specific enzymatic activities such as lamin-directed prolyl cis/trans isomerization or putative proteases involved in capsid-NEC interaction (e.g., HCMV pUL80), (iv) the direct interference with capsid docking to the NEC or cognate host factors of the NE, and (v) the blocking of membrane-specific activities of the NEC as a part of primary capsid envelopment. To evaluate these inhibitory strategies on a molecular-mechanistic level, a number of analysis systems have recently been reported for HCMV (Figure 7, Figure 8 and Figure 9) and other human herpesviruses [42,103,125]. An interesting point is that the recently approved anti-HCMV drug MBV/Livtencity^TM^ (Figure 9A), which represents the first prototype kinase inhibitor in the entire field of antiviral therapy, exerts a strong nuclear egress-inhibitory activity in vitro and in vivo [42,126,127]. This mechanistically new activity, i.e., the viral nuclear egress-specific mode of action (MoA), of this drug and further investigational candidates can be quantitatively assessed by applied test systems (Figure 9B). Combined, based on the novel achievements on recombinant herpesviral NEC protein expression, in particular by the use of hook::groove fusion constructs of recombinant core NECs [32] or, alternatively, through heterodimeric NEC copurification, their characterization, crystallization, structural analysis, in silico drug docking investigation and NEC-specific antiviral drug research, new ways have been explored. Thus, the respective strategies may hopefully provide a mechanistically new generation of antiviral drugs in the near future.

## Figures and Tables

**Table 1:** NEC conservation between herpesviruses.

Amino Acid Sequences	Poorly Conserved
3D structure of core NEC	Almost fully conserved
Principle of hook-into-groove interaction	Identical
Multicomponent interaction properties	In part conserved
Overall nuclear egress functionality	Conserved in final outcome
